# MoS_2_ Surface Structure Tailoring *via* Carbonaceous Promoter

**DOI:** 10.1038/srep10378

**Published:** 2015-05-21

**Authors:** Yumeng Shi, Henan Li, Jen It Wong, Xiaoting Zhang, Ye Wang, Huaihe Song, Hui Ying Yang

**Affiliations:** 1 Pillar of Engineering Product Development, Singapore University of Technology and Design, Singapore 487372; 2 Nanyang Technological University, School of Materials Science and Engineering, 50 Nanyang Avenue, Singapore 639798; 3 State Key Laboratory of Chemical Resource Engineering, Beijing University of Chemical Technology, Beijing 100029

## Abstract

Atomically thin semiconducting transition-metal dichalcogenides have been attracting lots of attentions, particularly, molybdenum disulfide (MoS_2_) monolayers show promising applications in field effect transistors, optoelectronics and valleytronics. However, the controlled synthesis of highly crystalline MoS_2_ remain a challenge especially the systematic approach to manipulate its structure and morphology. Herein, we report a method for controlled synthesis of highly crystalline MoS_2_ by using chemical vapor deposition method with carbonaceous materials as growth promoter. A uniform and highly crystalline MoS_2_ monolayer with the grain size close to 40 μm was achieved. Furthermore, we extend the method to the manipulation of MoS_2_ morphology, flower-shape vertical grown MoS_2_ layers were obtained on growth promoting substrates. This simple approach allows an easy access of highly crystalline MoS_2_ layers with morphology tuned in a controllable manner. Moreover, the flower-shape MoS_2_ grown on graphene oxide film used as an anode material for lithium-ion batteries showed excellent electrochemical performance.

Direct bandgap semiconducting transition metal dichalcogenide (TMD) monolayers are attractive for optoelectronics and energy harvesting^1^,^2^. Their high carrier mobility^3^, excellent on-off ratio^4^ and good bendability^5^ are promising for future flexible and low-power consumer electronics^5^,^6^. The fundamental studies on their electronic structures and valley-spin relations are also emerging^7^,^8^. Being a member of the TMDs family, molybdenum disulfide (MoS_2_) has already attracted considerable attention for its great potential in the fields of hydrogen evolution reaction (HER)^9^,^10^, bio-sensing^11^,^12^ and energy storage^13^,^14^,^15^. Recently, it has been demonstrated that ultra-thin MoS_2_ crystals with a typical thickness of ~0.65 nm holds even more significant promise in electronics^4^,^16^,^17^ and optoelectronics^7^,^18^,^19^,^20^,^21^,^22^.

Given the great promises hold, it is highly desirable to develop a scalable method to obtain large-area and high quality TMD monolayers. On the other hand, controlling the surface structure of MoS_2_ layers at the atomic scale is also critical for their successful implementation in each applications. For example, large specific surface areas are preferred for the energy storage application^13^,^14^. For the application of HER, only the edge sites of MoS_2_ are catalytically active, which require the controllable synthesis of MoS_2_ layers with abundant exposed edge sites^10^,^23^. Recently, chemical vapor deposition (CVD) method has led towards high-quality TMD layers with scalable size, controllable thickness and excellent electronic properties. Several groups have made remarkable progress in MoS_2_ synthesis^8^,^24^,^25^,^26^. Among the various approaches for MoS_2_ synthesis, those using MoO_3_ and sulfur powder as solid state precursors become dominant^26^,^27^,^28^,^29^,^30^, which is due to the simplified process and high yield of monolayers.

However, it is more challenging to grow single crystalline monolayer MoS_2_ thin film by CVD method comparing to other 2D materials synthesis such as graphene^31^,^32^ and boron nitride^33^,^34^, where catalyst were used to control the geometry, thickness and crystallinity. In contrast, the growth of MoS_2_ by CVD does not involve any catalysts^35^,^36^ and the growth of MoS_2_ is very sensitive to the substrate treatment prior to the growth^18^,^22^. Previous reports demonstrate nucleation of MoS_2_ can be facilitated by seeding the substrate with graphene-like species^29^,^30^,^37^. Meanwhile Najmaei *et al.* have shown that the MoS_2_ crystallines commonly nucleate on the step edges during growth without the present of seeding molecules^28^. Recently, Van der Zande *et al.* proved that large MoS_2_ crystalline islands can be obtained by using ultraclean substrates and fresh precursors and neither seeding molecules nor step-edge were used to promote the nucleation of MoS_2_^27^. It was suggested that the MoS_2_ growth normally follows an analogous layer-plus-island (or Stranski-Krastanov) growth mode^8^. The Stranski-Krastanov mode is a two-step process: initially, monolayer MoS_2_ domains gather and interconnect with each other till the full coverage of monolayer is nearly completed. Beyond the critical layer number (1 for MoS_2_), the growth of MoS_2_ continues through the nucleation and coalescence of MoS_2_ nanoparticles or few layers islands^8^. In other words, the thermodynamics favors the basal plane growth, which limits the tunability of surface structure.

Nevertheless, the widely used method for MoS_2_ synthesis is based on the direct chemical vapor phase reaction of MoO_3_ by S gas in which the MoO_x_ vapor generated from MoO_3_ powder reacts with sulfur vapor at elevated temperatures to form monolayer MoS_2_ on the collecting substrates^26^,^29^. Thus, it is common to obtain molybdenum oxide microcrystals byproducts since the reduction of MoO_3_ also produce MoO_2_ crystals under similar growth condition^38^. MoO_3_ was selected by Li *et al.* as an precursor for MoS_2_ growth on the basis that it has an evaporation temperature of ~700 °C^26^. However, the growth dynamics of MoO_3-x_ and S are still not fully understood. It has been suggested in literatures^39^,^40^ that there are two channels for MoO_3-x_ to react with S, one is it adsorb and diffuse on the substrate, reacting with S to form MoS_2_; the other is forming MoS_2_ clusters in vapour phase and crystalize on substrate. Both these two channels require the forming of vapour phase MoO_3-x_; and the growth temperature ranging from 530 °C to 850 °C. Thus far, it is demanding to have a better understanding of the growth mechanism and further develop a method which is capable of producing large monolayer MoS_2_ crystals, preventing the formation of MoO_2_ byproducts, meanwhile controlling the surface structure of MoS_2_ layers at the atomic scale.

Herein, we engineer the reduction process of MoO_3_ precursor by carbon based materials. In a typical synthesis, the solid state precursor of MoO_3_ powder is covered by a piece of carbon cloth. The carbon cloth is chosen simply due to its thermal stability, good mechanical property and micro porous structure. MoO_3_ powder can be thermally evaporated and only the gas phase MoO_3_ can pass through the opening of woven micro carbon fibers and it is then reduced by sulfur vapor at 650 to 700 °C. It was found that carbon can help reducing MoO_3_ and large MoO_2_ crystals can be trapped by the carbon cloth which effectively prevents the co-deposition of MoO_2_ and MoS_2_ on the collecting substrates. Finally, continuous large-area MoS_2_ thin films was successfully grown on substrate. The synthesized MoS_2_ layers are in a well-defined triangular shape with a typical lateral size ~40 μm. We further investigated the effects of carbonaceous promoter in the growth of MoS_2_ on various substrates. By simply replacing the SiO_2_/Si substrates with GO film or GO flakes, few-layer MoS_2_ preferentially form on the surface of GO under the same growth condition. The results suggest, the growth of MoS_2_ is very sensitive to the substrate treatment and carbon based materials can significantly promote the growth rate and yield of MoS_2_, which is due to the assisted reduction of MoO_3_ by carbon.

This work elucidates how morphological control of MoS_2_ at the nanoscale can be achieved by carbonaceous promoter. The surface morphology engineering of MoS_2_ layers enables new opportunities for enhancing surface properties for catalysis, energy storage and other important technological applications. As a proof-of-concept, the MoS_2_/GO composites were used as electrode materials to demonstrate its application in lithium ion batteries (LIBs). The surface structure engineering of MoS_2_/GO provides highly efficient pathways for both electronic and Li ion exchange during the charge/discharge cycles of the battery, which allows the composite to be directly used as working electrode and assembled into a coin cell without adding any conductive or binder materials. A remarkably high specific capacity (i.e., > 1000 mAh g^−1^) was achieved at the current density of 100 mA g^−1^, which is much higher than theoretical value for either GO or MoS_2_ alone (~566 and ~670 mAh g^−1^, respectively). The MoS_2_/GO composites also show an outstanding high-rate charge/discharge performance. Even at a very high current density of 1000 mA g^−1^, the composite electrode can still deliver a capacity of 776 mAh g^−1^ after 500 cycles. The reversible capacity only slightly decreases to 727 mAh g^−1^ after an additional 440 cycles under 2000 mA g^−1^. The high rate capability can be attributed to the unique nano-architecture engineering of MoS_2_, which provides structural stability and transport advantages for both electrons and lithium ions.

## Materials and Methods

### Monolayer CVD-MoS_2_ growth

CVD-MoS_2_ was prepared using chemical vapor deposition method with carbon based materials as a growth promoter. High-crystal-quality MoS_2_ can be grown on a silicon substrate with 300 nm SiO_2_ layer on top inside a hot-wall horizontal tube furnace. To be brief, the MoS_2_ films were synthesized using high purity MoO_3_ (99%, Aldrich) and S powder (99.5, Sigma-Aldrich) as precursors. The precursors were placed in two separated Al_2_O_3_ crucibles and the substrates were placed on the downstream side of the Ar carrying gas. A piece of carbon cloth was put on top of MoO_3_ powder for better growth control. The growth chamber was firstly heated to 105 °C with Ar flow rate of 1000 sccm, this step helps to remove the oxygen and moistures in the chamber. After that, the temperature was further increase to 700 °C with a heating rate of 15 °C/min. MoS_2_ monolayer in a triangle shape were obtained by annealing at 700 °C for 10 min followed by a naturally cooling process to room temperature Ar flow rate was kept at 10 sccm during MoS_2_ growth.

### Flower shape MoS_2_ growth

Before the CVD synthesis, 1 mg/ml Graphene oxide (GO) dispersion in water was obtained after 30 min probe sonication of GO flakes (Graphene Supermarket, USA.) in deionized water (DI Water). The 300 W probe sonicator was set at 30% amplitude with alternating pulse. GO coated Si substrate was prepared by drop casting GO dispersion on a piece of cleaned SiO_2_/Si substrates and gently blow dried using N_2_ gas. Self-supporting GO thin film was obtained by vacuum filtration of GO dispersion with a polymer filter membranes (pore size 0.02 um, GE Whatman, USA). The filtration membrane was further removed by dissolving it in hot Acetone solution (at ~80 °C). The GO substrates were carried into the growth chamber after baking on hot plate at 90 °C for 1 hour to remove organic solvent and water. The growth condition was kept the same as monolayer MoS_2_ synthesis.

### Chemicals and precursors

Graphene oxide powder was purchased from Graphene Supermarket, Calverton, NY, USA. MoO_3_ (purity 99%) and S powder (purity 99.5%) powder purchased from Sigma-Aldrich Co. (Singapore) were directly used for MoS_2_ synthesis without further purification. Carbon cloth was purchased from Hesen Shanghai Co., Ltd, China.

### MoS_2_/GO transfer for TEM characterization

The CVD synthesized MoS_2_/GO composite was transferred by coating the film with a thin layer (~100 nm) of Poly[methylmethacrylate] (PMMA). After etching the underlying SiO_2_/Si substrates with NaOH aquariums (with a concentration of 3 M) at 80 °C, the PMMA/ MoS_2_/GO film was transferred to DI water and was suspended on the surface of water to remove the etchant residue for several hours. Subsequently, the film can be transferred to any substrate or TEM grids for analysis and characterization. Finally, the top layer of PMMA can be removed by acetone or by directly annealing the samples in an Ar and H_2_ atmosphere at 450 °C for 2 hours.

### Raman measurements

The Raman measurements were carried out using a WITec alpha 300 confocal Raman microscope. The Raman spectra presented in this paper were collected using a 532 nm solid-state laser for excitation with the beam focused by a 100X objective lens. The laser beam diameter on sample is around 500 nm. Scanning electron microscopy (SEM) was performed on a field emission SEM (FESEM) instrument (JSM-7600F, Japan). A field-emission transmission electron microscope (JEOL JEM-2100F, operated at 200 keV), equipped with an energy dispersive spectrometer (EDS) was used to obtain the information of the microstructures and the chemical compositions.

### Electrochemical measurement

The electrochemical performance of MoS_2_/GO nanocomposites electrode was measured with a half-cell lithium ion battery (LIBs) configuration. The 2032 coin-type cells were assembled in an argon-filled glove-box with both the moisture and oxygen level less than 0.5 ppm. MoS_2_/GO composites were directly used as cathodes. For control samples, the working electrode materials of GO and MoS_2_ were prepared by mix GO and MoS_2_ powders with a certain weight percentage in solution phase and then freeze-dried to form a self-supporting membrane. Lithium sheet was used as anodes and 1 M LiPF6 in a 1/1 (volume ratio) mixture of ethylene carbonate (EC)/dimethyl carbonate (DMC) as electrolyte. Celgard® 2400 was used as the separator of the battery. The cells were tested on a NEWARE multi-channel battery test system with galvanostatic charge and discharge in the voltage range between 0.01 and 3.0 V vs. Li/Li^+^ at various current density at room temperature. The cyclic Voltammetry (CV) and electrochemical impedance spectroscopy (EIS) were tested on an electrochemical workstation (VMP3, Bio-Logic).

## Results and Discussion

### Effect of carbon based materials on monolayer MoS_2_ growth

**Figure 1:**
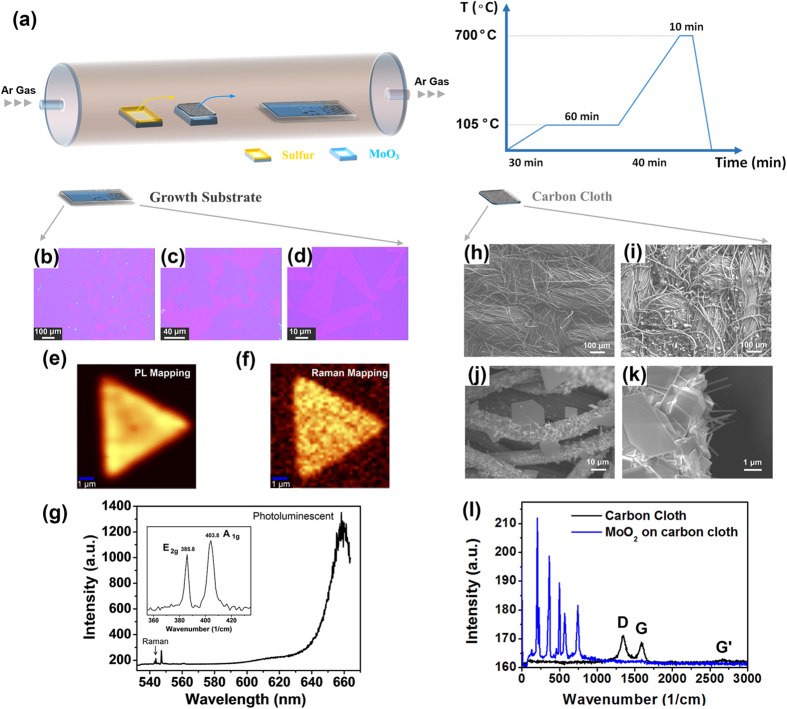
(**a**) Schematic illustration of the experimental set-up for CVD-growth of MoS_2_; and illustration of temperature profile for the MoS_2_ growth process at different stages. (**b**), (**c**) and (**d**) show the optical images for as-grown MoS_2_ monolayer films and isolated monolayer crystallites. (**e**) Photoluminescence (PL) map of the synthesized MoS_2_ monolayers, and (**f**) shows the corresponding Raman intensity map. The synthesized MoS_2_ were grown on SiO_2_/Si substrates. (**g**) Experimental results show the PL spectrum of MoS_2_ films. Inset, typical Raman spectra collected from the area of the MoS_2_ monolayer film. (**h**) and (**i**) SEM images of carbon cloth surface before and after CVD process; (**j**) and (**k**) SEM images show large crystals can be found on carbon cloth surface; (**l**) Raman spectrum taken from the pristine carbon cloth and the one after CVD synthesis.

A schematically illustration of the experimental set-up used for MoS_2_ synthesis is presented in Fig. 1 (a). MoS_2_ monolayers were grown by CVD with solid MoO_3_ and S precursors. In contrast to previous reports, the MoO_3_ powder with a weight of ~15 mg was directly placed on a silicon wafer which is next to the collecting substrates for MoS_2_ growth. A piece of carbon cloth (thickness: 0.34 ± 0.02 mm, surface area ~50 mm^2^) was put on the top of MoO_3_ powder. Prior to the growth, argon gas was used to flush the quartz tube thoroughly in order to remove the oxygen and moistures. 10 sccm of argon was supplied during the synthesis of MoS_2_ monolayers, while the growth chamber was heated from room temperature to 700 °C with a temperature profile as shown in Fig. 1 (a).

Detailed growth procedure can be found in the method section. At such temperature, MoO_3_ powder evaporated and reacted with sulfur vapor to form a continuous MoS_2_ films and isolated triangular MoS_2_ domains can also be found at the edges along the film which are shown in the optical microscopy images (with different magnifications) of Fig. 1 (b), (c) and (d) where the MoS_2_ sheets were grown on the SiO_2_/Si substrate. The triangular shape of the crystallites reflects the 3-fold symmetry of MoS_2_ that suggests they are single-crystalline^30^,^41^. Similar to previous work, the monolayer MoS_2_ can be merged to form a large MoS_2_ sheet^29^, and among different growths the average size of MoS_2_ islands varies between 10 to 40 μm. The intensity of photoluminescence (PL) peak and the energy separation between the Raman A_1g_ and E_2g_ peaks have been found related to the number of MoS_2_ Layers^42^,^43^,^44^. Therefore, Raman and PL measurement were carried out to confirm the quality of the individual crystallites. Fig. 1 (e) and (f) show the PL intensity mapping and the corresponding intensity mapping of Raman peak of an isolated triangular MoS_2_ crystallite. Figure 1 (g) displays the typical spectra taken from the MoS_2_ crystallite that consisting both the Raman and PL peaks. The strong PL peak and high PL to Raman intensity ratio suggest the direct band gap photoluminescence. The inset figure in Fig. 1 (g) presents the Raman peak of monolayer MoS_2_, the E_2g_ and A_1g_ peaks locate at 385.8 and 403.8 cm^−1^, respectively with a peak distance of 18 cm^−1^. The small E_2g_ and A_1g_ peak distance suggests the monolayer nature of these MoS_2_ crystallites^45^,^46^.

We note that without the carbon cloth, triangular shape MoS_2_ can also be obtained but there will be few-layer MoS_2_ and/or MoO_2_ crystallites distributed among them, as shown in Supporting Data (Fig. S1). As a consequence, under our growth condition, carbon based materials play an important role in facilitating the monolayer MoS_2_ growth. It is worthy to mention that, recently reports demonstrated aromatic molecules are helpful for the nucleation of the MoS_2_ layers^29^,^30^. However, it is still controversial that a larger single layer MoS_2_ can be obtained by using carefully cleaned substrates^27^,^28^. In this study, the carbon cloth is separated from the collecting substrates, and pre-annealed at 700 °C to exclude the influence of any impurities from the carbon cloth. It is generally believed that at elevated temperatures the MoO_x_ vapor generated from MoO_3_ powder reacts with sulfur vapor and being sulfurized to form monolayer MoS_2_ on the collecting substrates, therefore the reduction of MoO_x_ is a critical step for the MoS_2_ formation^38^.

Accordingly we further investigated the surface reaction of carbon cloth with MoO_x_ during MoS_2_ synthesis. Figure 1 (h) and (i) show the Scanning Electron Microscope (SEM) images of the carbon cloth surface before and after the growth. The pristine carbon cloth is manufactured in bundles of thousands of tiny fibers, and woven onto a fabric roll. After growth, the surface of carbon cloth facing the MoO_3_ precursor are fully covered by micro size flakes or large crystals, as displayed in Fig. 1 (j) and (k). Figure 1 (l) compares the Raman spectrums from carbon cloth before and after the CVD process. These micro crystals deposited on the carbon cloth surface show strong Raman peaks and the carbon peaks at around 1300 and 1600 cm^−1^ become comparatively weaker. According to the literature, the additional Raman peaks obtained after CVD process belongs to MoO_2_^47^,^48^. However, there are no detectable MoS_2_ Raman peaks from carbon cloth.

The forming of MoO_2_ crystals on the surface of carbon cloth suggests carbon is more reductive under elevated temperature to react with vapor phase MoO_3_. In our experiments, the carbon cloth directly contacted with MoO_3_ powder, thus no sufficient sulfur vapor can penetrate through the carbon layer to react with the precursor underneath. In other words, the additional carbon layer helped to create an abrupt MoO_3-x_ concentration change between the top and bottom layer of the carbon cloth. It is well know that the reduction of MoO_3-x_ by sulfur can produce MoO_2_ and further sulfurization gives MoS_2_ layers^27^,^29^,^30^,^37^. Therefore, it is likely that during the growing process where the carbon layer provides a more steady and sustainable flow of MoO_3-x_ (x>1) and results a more constant ratio of Mo and S to form stoichiometry MoS_2_ crystallites. The reduction capability of carbon cloth was also compared with other form of carbon such as well crystallized highly ordered pyrolytic graphite (HOPG). Our results suggest well crystallized carbon, such as HOPG is more inert in the reaction with MoO_3_ during the growth (See the Supporting Data). The Raman spectra in Fig. 1 (l) show a strong D band at around 1300 cm^−1^, which indicates the defective nature of carbon cloths. The defective nature of carbon cloth makes it more reactive and attractive for molybdenum source with a large interacting surface with MoO_3-x_. Thus these carbon fibers assist the reduction of MoO_3-x_ for MoS_2_ growth with improved reactivity and efficiency.

Recently, Kong *et al.* reported vertically aligned MoS_2_ and molybdenum diselenide (MoSe_2_) layers can be produced by a rapid sulfurization/selenization process at 550 °C^49^. It is suggested that the formation of vertically aligned TMD layers is driven by a kinetic process. When the growth is limited by the diffusion of sulfur/selenium, due to the anisotropic structure of TMD layers, it is much faster for sulfur/selenium to diffuse along the van der Waals gaps. Therefore, the TMD layers naturally oriented perpendicular to the film, exposing van der Waals gaps for fast reactions^49^. The assisted reduction process of MoO_3-x_ by carbon based materials is likely to alter the growth rate and thus provides a way to tune the surface structure of TMD layers.

**Figure 2:**
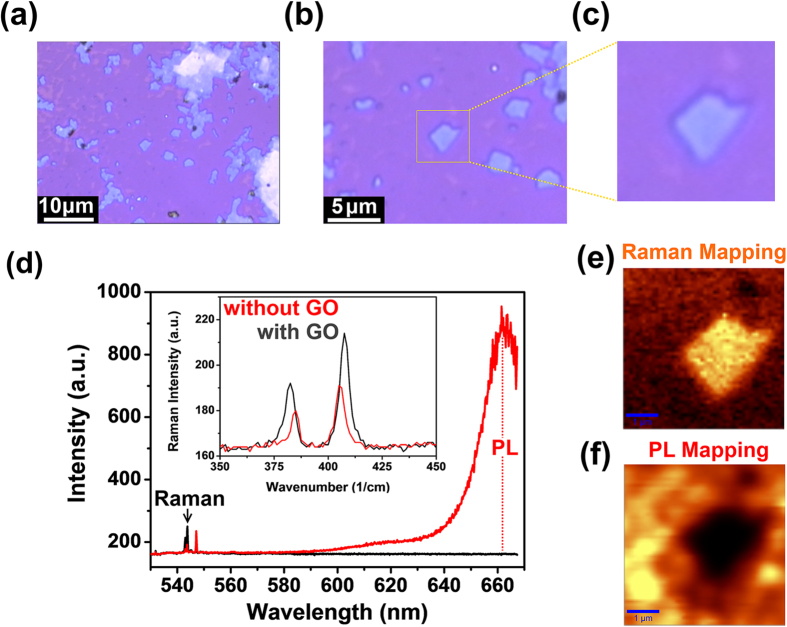
(**a**), (**b**) and (**c**) present the optical microscope images of GO coated SiO_2_/Si substrates after CVD synthesis taken under various magnifications. (**d**) Typical PL and Raman spectrums taken from monolayer and few layer MoS_2_ regions. (**e**) and (**f**) are the corresponding Raman and PL maps in Fig. (**c**), the rectangular shape present a few layer MoS_2_ with monolayer MoS_2_ surrounding it.

In order to better understand the growth mechanism and further develop a method which is capable of engineering the surface structure of MoS_2_ layers in a controlled manner. Carbon based materials were deposited directly on the growth substrates to tune the local growth condition. Graphene oxide (GO) prepared by modified Hummers method is generally accepted as a defective form of carbon and GO flakes with abundant function groups also show similar Raman feature with the carbon cloth used in our study (See Supporting Data). Therefore, GO was chosen to reveal the reaction between carbon and MoO_3-x_ and its effects on the crystallization of MoS_2_ crystallites. To investigate the growth mechanism and reveal the role of carbon promoter during MoS_2_ formation, GO dispersed in DI water was drop casted on cleaned Si wafer with 300 nm SiO_2_ top layer. The GO casted substrates were carried into CVD furnace and MoS_2_ growth was then carried out. Figure 2 (a), (b) and (c) show the optical image of GO coated SiO_2_/Si wafer after growth. The color contrast is due to the different layer thickness of deposited materials. Flakes with irregular shape can be found among the single layer MoS_2_. We chose a location that fully covered with MoS_2_ monolayer and GO flakes can be found distributed among the mono layer MoS_2_. The Raman measurement clearly shows the distribution of monolayer and few-layer MoS_2_. Spectrum in Fig. 2 (d) shows a typical comparison of PL and Raman peak intensity taken from an irregular shaped few layer MoS_2_ and its surrounding. Note that the Raman peak of GO becomes very weak after CVD synthesis. The mapping in Fig. 2 (e) and (f) displays the peak intensity distribution on the sample surface. Moreover, it is worthy to mention that for the typical growth, most of the SiO_2_/Si surface were covered with triangular monolayer MoS_2_ crystallites or MoS_2_ film. Meanwhile, all the MoS_2_ Raman peaks taken from the GO surface have a large E_2g_ to A_1g_ peak distance suggesting they are few-layers (see supporting information). These results are solid evidence that the GO tends to attract and promote MoS_2_ growth. We also noticed that without sulfur supply, monolayer GO is more reactive and can be totally etched away by MoO_3_ vapor (see supporting information) which also suggests the carbon MoO_3_ reduction reaction takes place during the growth.

### Flower-shape MoS_2_ growth, characterization and application

**Figure 3:**
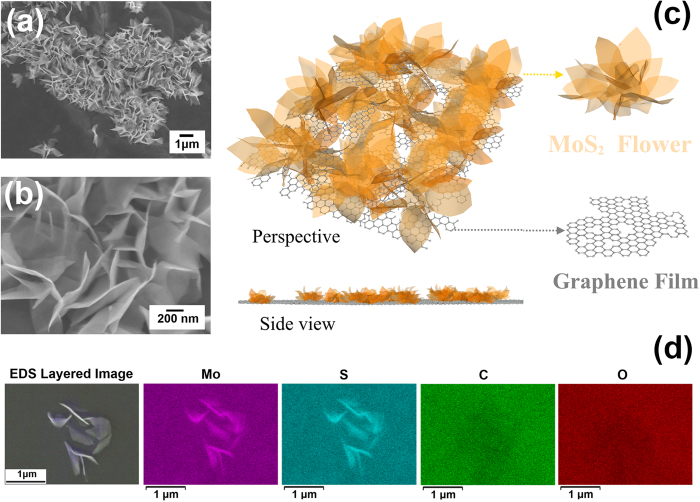
(**a**) and (**b**) show the SEM images of samples obtained by using GO thin film as growth temples. (**c**) Schematic illustration of flower-shape MoS_2_ grown on GO thin film. (**d**) EDS mapping of a layered structure region. The EDS intensity maps confirm the chemical composition of these layered structures to be MoS_2_.

Since it was reported that sulfurization process largely affects the layer orientations in the synthesized chalcogenide^49^,^50^,^51^. As discussed, at high temperatures, carbon can dramatically enhance the reduction of MoO_3_, thus promote the formation of MoO_3-x_ (x>1) to react with sulfur vapor which further converts to MoS_2_ layers. Thereby under monolayer MoS_2_ growth condition, the introduction of additional carbon materials is likely to create a localized sulfur diffusion limited process for MoS_2_ growth. In order to investigate the effect of carbon on the layer orientations of synthesized chalcogenides, we intentionally apply more carbon based materials by utilizing filtrated GO thin film as the growth template, and all the other growth conditions were kept unchanged. The as grown samples possess a dramatically different morphology as shown in Fig. 3 (a) and (b). Interestingly, the synthesized MoS_2_ layers tend to form in a micrometer flower shape as illustrated in Fig. 3 (c). Figure 3 (d) displays the energy-dispersive X-ray spectroscopy (EDS) mapping characterization, which confirms the chemical composition of the flower shape structure.

**Figure 4:**
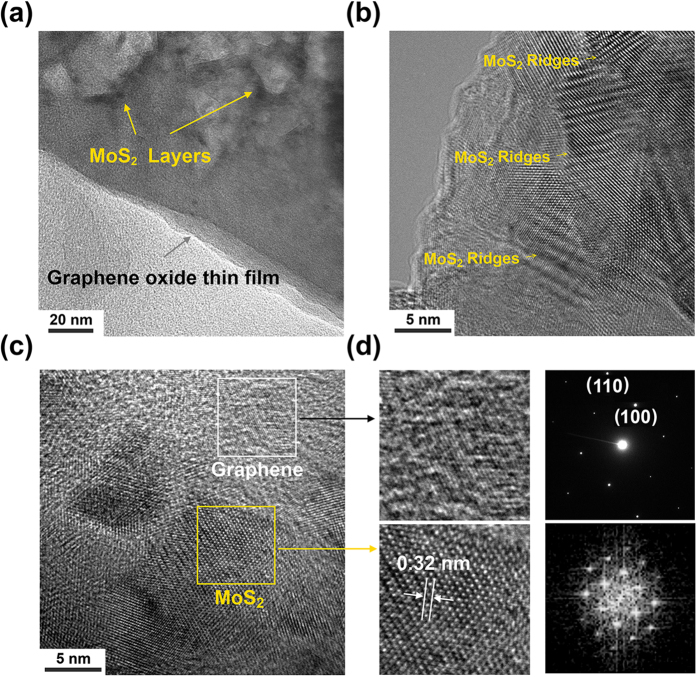
(**a**) and (**b**) TEM images of MoS_2_/GO composite under different magnifications; (**c**) HRTEM image shows both graphene and MoS_2_ regions; (**d**) enlarged HRTEM images display the corresponding carbon and MoS_2_ lattice structures. SAED in upper right confirms the back ground is graphene. FFT in the bottom right, suggests the hexagonal arrangement of S-Mo-S elements in MoS_2_ layers.

Further structural characterizations using transmission electron microscopy (TEM) provide additional insights into MoS_2_/GO composite film. Figure 4 shows the typical TEM images of MoS_2_/GO film. Figure 4 (a) displays a low magnification TEM image, where the MoS_2_ layers show a darker color contrast on GO thin film. Figure 4 (b) exhibits a high-resolution TEM (HRTEM) image taken along the edge part of these composites. Stripe-like grains with ~10 nm in length and several nanometers in width were found, however these grains are densely packed and overlapped with each other preventing an accurate lattice structure analysis. In Fig. 4 (c), the HRTEM image on a single grain reveals individual atomic planes ordered in the S–Mo–S sequence to form each layer. The carbon surface with a lighter color contrast under TEM was confirmed to be graphene by performing the selected area electron diffraction (SEAD) and the FFT confirms the hexagonal arraignment of S-Mo-S atoms. As shown in Fig. 4 (d), both the HRTEM image and fast Fourier transform (FFT) pattern reveal that MoS_2_ flakes grown on GO retain the crystal symmetry with the lattice constant ~0.32 nm. Both of the EDS and TEM analysis suggests the MoS_2_ layers tend to form a layered flower-shape structure on GO substrates compared to monolayer in plan growth on SiO_2_/Si substrates. The morphology change is likely due to the promoted conversion rate of MoO_3-x_, where the chemical conversion occurs much faster than the diffusion of sulfur gas into the film, forcing the MoS_2_ layers naturally oriented perpendicular to the growth substrates, exposing van der Waals gaps for fast reaction^49^.

**Figure 5:**
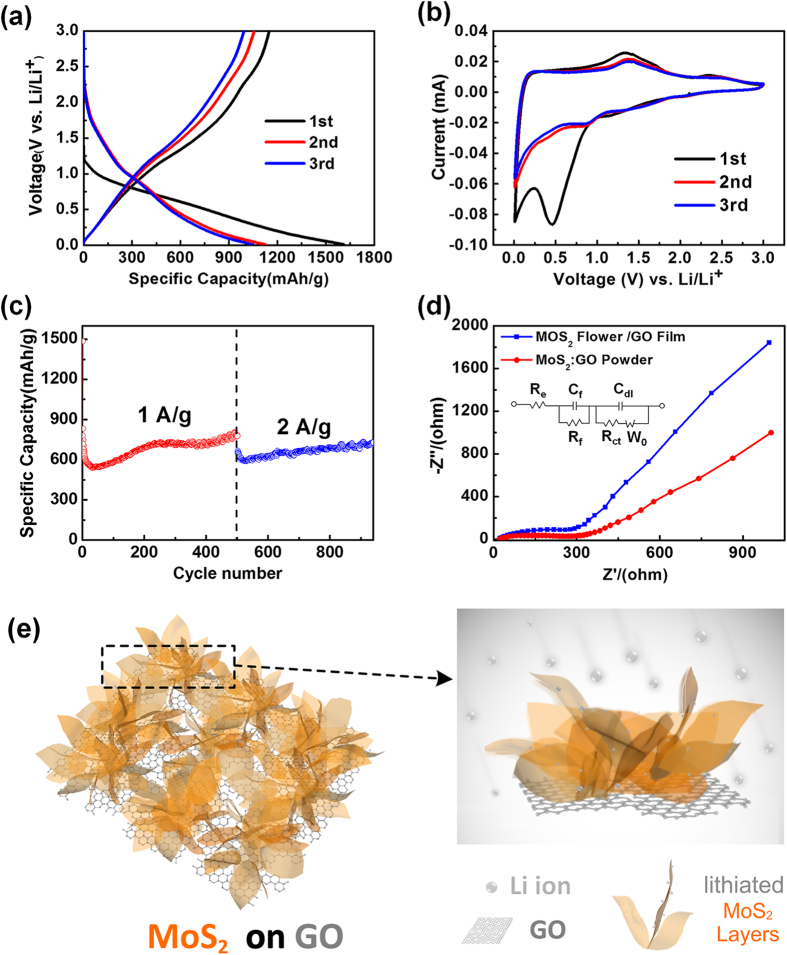
(**a**) Charge-discharge curves of MoS_2_/GO composites at current density of 100 mAg^−1^; (**b**) Representative cyclic voltammograms of MoS_2_/GO composite at the first 3 cycles between 0.01 V and 3.0 V at scan rate of 0.1 mV s^−1^; (**c**) The cycling performance of MoS_2_/GO composite at various current densities of 1 A g^−1^ and 2 A g^−1^ (**d**) Nyquist plots of MoS_2_/GO composite and MoS_2_:GO powder blended electrode after 10 discharge/charge activation cycles. Inset shows the equivalent circuit model used for the fittings and the corresponding values are shown in Table 1. (**e**) Schematic illustration of the surface structure of MoS_2_/GO composite for high current density lithium storage.

As a proof-of-concept application, the electrochemical property of the MoS_2_/GO composite as an anode material of a Lithium ion battery (LIB) was further investigated by galvanostatic discharge/charge and cyclic voltammetry (CV) measurements. The measurement was based on a half-cell configuration as shown in the supporting materials. Figure 5 (a) illustrates the first, second and third discharge/charge voltage profiles of the flower shape MoS_2_/GO composite electrode grown by CVD method. The test were carried out in the voltage range from 0.01 to 3 V (vs. Li/Li^+^). The initial discharge and charge capacities were measured to be 1612 and 1149 mAh g^−1^, respectively, with a corresponding initial Coulombic efficiency of 71.3%. The irreversible capacity loss for the first cycle could be mainly attributed to the electrolyte decomposition and inevitable formation of the solid electrolyte interface (SEI), which are commonly observed for nanosized anode materials^13^. The second and third cycles show a decreased capacity but with a much higher Coulombic efficiency of 93.8% and 95.3%, respectively. In the first discharge process, the initial discharge capacity between 2.0 to 1.5 V can be attributed to the reaction of residual carbon surface functional group and the lithium insertion to MoS_2_ which forms Li_x_MoS_2_. The following capacity between 1.0 to 0.5 V can be attributed to the conversion reaction process of MoS_2_, where the metal sulfide reacts with lithium ions forming metal nanoparticles and insoluble Li_2_S matrix, MoS_2_+4Li^+^+4e^−^→Mo+2Li_2_S^14^. The formation of a SEI and the gel-like polymeric layer on the surface of the active materials contribute to the sloping plateau at the lower voltage region (< 0.5 V). In the charge process, the plateau at ~1.3 V and the sloping region above 2.2 V can be attributed to the reverse reaction process, where oxidation of Mo particles to MoS_2_ and the conversion of Li_2_S to S and Li^+^ occur^14^,^52^,^53^. It is noted that the lithium extraction from the Li_x_MoS_2_ phase should also be considered.[Table t1]

To further clarify the electrochemical process of MoS_2_/GO composite, cyclic voltammogram (CV) measurements of the first three cycles in the voltage range of 0.01-3.0 V at a scan rate of 0.1 mV s^−1^ were carried out as shown in Fig. 5 (b). In the initial cycle, two reduction peaks at 1.1 and 0.5 V were observed, which can be attributed to the formation of Li_x_MoS_2_ and the conversion reaction that leading to Mo metal nanoparticles embedded in a Li_2_S matrix. In the reverse anodic scan, the oxidation peak at ~ 1.4 V can be partly attributed to the oxidation of Mo to MoS_2_ and the peak at ~ 2.3 V can be assigned to the oxidation of Li_2_S to S. In the 2^nd^ CV scan, the reduction peak at 0.9 V can be attributed to the Li insertion into MoS_2_ lattice to form Li_x_MoS_2_. The weak reduction peak at ca. 2.1 V indicates the formation of Li_2_S. In the anodic sweeps, the oxidation peaks at 1.4 V and 2.3 V correspond to the extraction of Li^+^ from Li_x_MoS_2_ lattice and the oxidation of Li_2_S, respectively. The results are in agreement with the above lithiation and delithiation profiles.

The MoS_2_/GO composites also possess outstanding high-rate performance. Figure 5 (c) shows cycle performance of the composite electrodes under high current density of 1000 and 2000 mA g^−1^. The initial discharge and reversible capacities are ca. 1484 and 779 mAh g^−1^, respectively, at 1000 mA g^−1^, which retain ca. 68% of capacities at 100 mA g^−1^ (see supporting information). After the initial 36 cycles, the specific capacity slightly decreased to 542 mAh g^−1^. However, a subsequent increase of specific capacity was observed for MoS_2_/GO composites, which should be attributed to the gradual activation of the electrode during lithiation and delithiation and a formation of gel-like film resulting from decomposition of the electrolyte at a low voltage. Due to the vertical structure of MoS_2_, the Li^+^ could get sufficient contact with the atomic layers of MoS_2_. As a result, excellent electrochemical performance was achieved for vertical grown MoS_2_ and GO film. The reversible capacity can retain 776 mAh g^−1^ even after a long cycling period of 500 cycles. When the current density increases to 2000 mA g^−1^, the reversible capacity after 940 cycles can still retain ~727 mAh g^−1^, which is about 94% of that at 1000 mA g^−1^.

The MoS_2_/GO composites electrodes were further investigated by the electrochemical impedance spectroscopy measurement where the Nyquist plots of MoS_2_/GO composite and MoS_2_:GO (commercial MoS_2_ and GO powder mixed in solution phase followed by freeze-dried to form a self-supporting film) powder blended electrode after 10 discharge/charge activation cycles are shown in Fig. 5 (d). The corresponding equivalent circuit is shown in the inset of Fig. 5 (d). The measurement indicates that the film resistance (R_f_) and charge-transfer resistance (R_ct_) of MoS_2_/GO are ca. 27 and 112 Ω, respectively. Both the R_f_ (106 Ω) and R_ct_ (130 Ω) of MoS_2_:GO mixture are much larger than those of MoS_2_/GO electrode. Since the MoS_2_/GO composites electrode possesses lower charge-transfer resistance, the charge transfer is lower than that of MoS_2_: GO blends. The significantly improved charge capacity of MoS_2_/GO composites could be attributed to the unique nanostructure of MoS_2_ sheets on the surface of GO film. The vertical structured MoS_2_ with extruding layers on GO has a much larger surface area, which provide more active sites during charging-discharging processes. In addition, lithium ions can be inserted/extracted into/out-of the vertical MoS_2_ flakes from both sides of MoS_2_, leading to a quick lithiation and delithiation process even under a large current density, as shown in the schematic diagram in Fig. 5 (e). Therefore, the MoS_2_/GO composite electrodes show excellent rate performance.

## Conclusion

In conclusion, we propose a carbon promoted process to synthesize large-area and highly crystalline MoS_2_ thin layers. The addition of carbon based materials during the high-temperature annealing drastically enhances the reduction of MoO_3_ vapor, as evidenced by various spectroscopic and microscopic characterizations including Raman, PL, TEM, and SAED. In particular, using GO thin film as the growth template results in an edge-terminated layered chalcogenide films forming in a flower shaped MoS_2_/GO composite. These MoS_2_ thin layers tend to orientate perpendicular to the growth substrate due to the sulfur diffusion limited process. The synthetic approach is simple and scalable, providing not only an easy but also efficient way to manipulate the structure of chalcogenide films. The unique structure paves the way to use the edges of layered materials more effectively.

## Additional Information

**How to cite this article**: Shi, Y. *et al*. MoS_2_ Surface Structure Tailoring *via* Carbonaceous Promoter. *Sci. Rep.* **5**, 10378; doi: 10.1038/srep10378 (2015).

## Supplementary Material

Supporting Information

## Figures and Tables

**Table 1 t1:** Fitting results of the EIS curves in Fig. 5 (d) using the equivalent circuit.

Sample	**R_s_ (Ω)**	**R_f_ (Ω)**	**R_ct_ (Ω)**
MoS_2_:GO powder	28	106	130
MoS_2_ flower/GO film	19	27	112
