# Corrigendum: MoS_2_ Surface Structure Tailoring *via* Carbonaceous Promoter

**DOI:** 10.1038/srep19323

**Published:** 2016-01-20

**Authors:** Yumeng Shi, Henan Li, Jen It Wong, Xiaoting Zhang, Ye Wang, Huaihe Song, Hui Ying Yang

Scientific Reports 5: Article number: 10378; 10.1038/srep10378 published online: 05212015; updated: 01202016.

The Acknowledgements section is incomplete.

“This work was supported by SUTD-MIT international design center (IDC) grant.”

should read:

“This work was supported by SUTD-MIT international design center (IDC) grant and SUTD Temasek Seed funding (IGDSS1402031).”

