# Supplementary material for: MoS2 Surface Structure Tailoring via Carbonaceous Promoter
Source: Sci Rep. 2015 May 21;5:10378. doi: 10.1038/srep10378 (PMC4650809; doi:10.1038/srep10378)
Supplement: Supporting Information [file srep10378-s1.pdf]

MoS_2 Surface Structure Tailoring *via* Carbonaceous Promoter

Yumeng Shi,[†,#] *Henan Li,* [┴,#] *Jen It Wong,*[†,#] *Xiaoting Zhang,*[^] *Ye Wang,*[†]
Huaihe Song,[^] *Hui Ying Yang,*[†,*]

[†] Pillar of Engineering Product Development, Singapore University of Technology and Design, Singapore 138682

[┴] Nanyang Technological University, School of Materials Science and Engineering, 50 Nanyang Avenue, Singapore 639798

[^] State Key Laboratory of Chemical Resource Engineering, Beijing University of Chemical Technology, Beijing 100029

[*] To whom correspondence should be addressed. E-mail: (H.Y. Yang) yanghuiying@sutd.edu.sg;

# These authors contribute equally

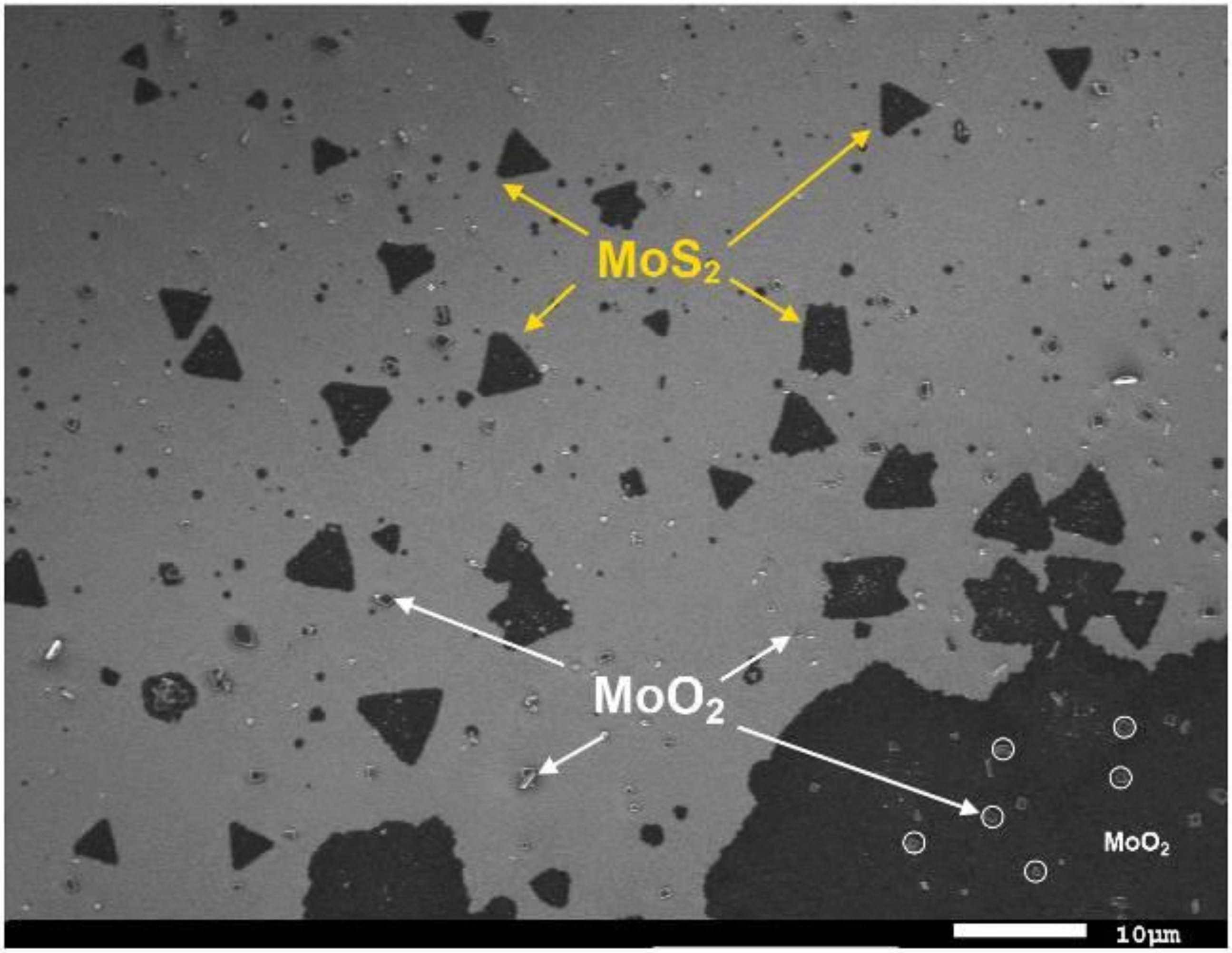

Figure S1 SEM image of CVD MoS_2 growth without using carbon cloth. Monolayer MoS_2 layers can be found along with MoO_2 crystallites which are indicated by the yellow and white arrows. The distribution of both MoS_2 and MoO_2 are random. It can be seen that MoO_2 crystallites with various size stack on the surface of monolayer MoS_2 or large size MoS_2 films formed by merged monolayer MoS_2 crystallites.

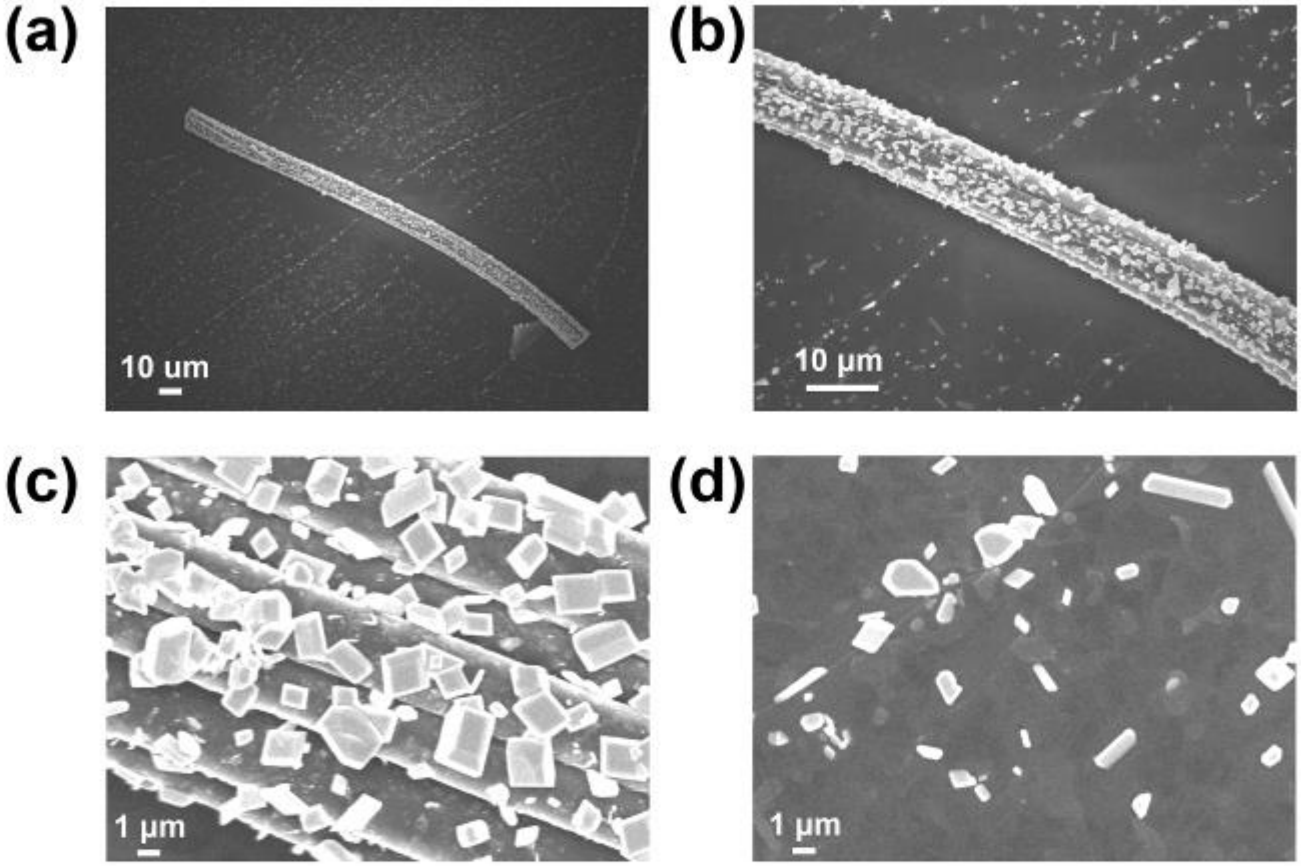

Figure S2 Comparison of SEM images of HOPG and carbon fiber (peeled from carbon cloth) after CVD synthesis. (a) an isolated carbon fiber on HOPG; (b) magnified SEM image shows more MoO_2 crystallites on carbon fiber surface; (c) zoomed in SEM image shows micro size MoO_2 crystallites grown on carbon fiber; (d) MoO_2 crystallites found on HOPF surface.

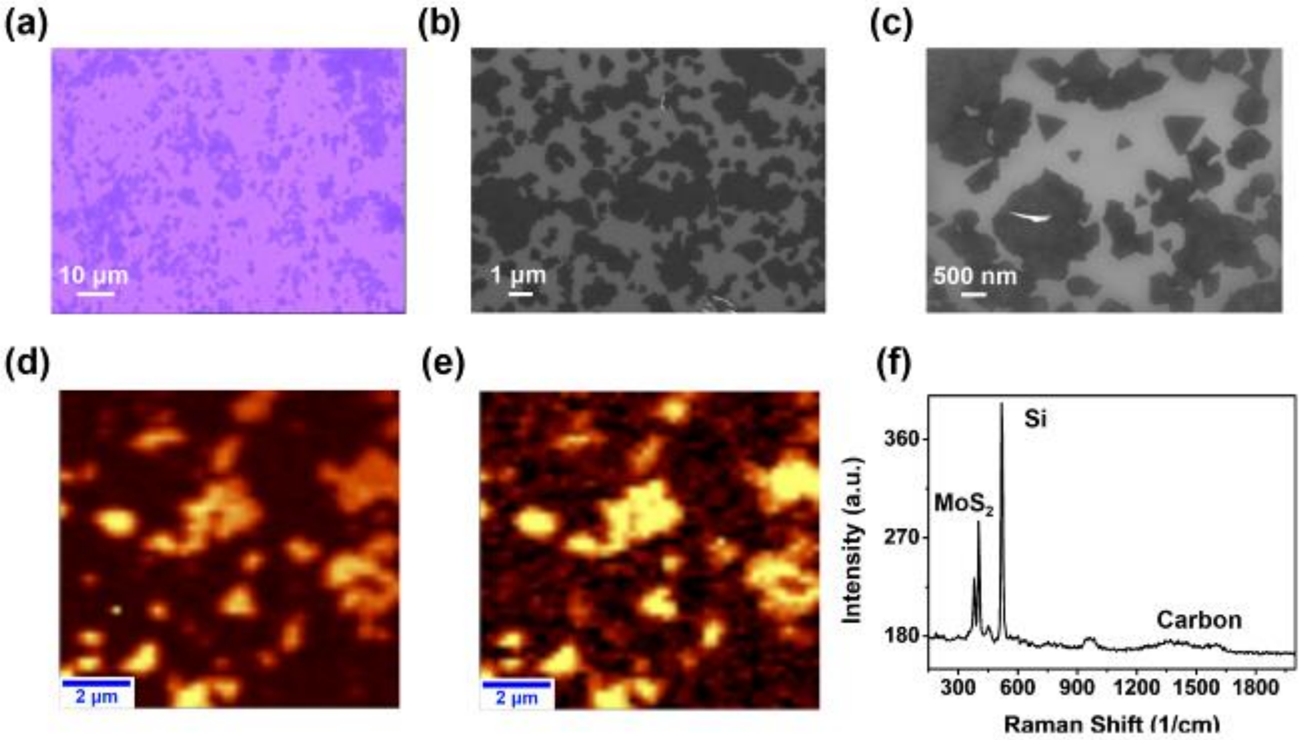

Figure S3 MoS_2 growth on GO flakes drop casted on SiO_2/Si substrates. (a) optical microscope image of GO drop casted Si wafer; (b) and (c) SEM images of MoS_2 growth on GO drop casted substrates; both triangle monolayer MoS_2 and few layer MoS_2 with irregular shape can be found; (d) and (e) Raman intensity mapping of carbon and MoS_2 peaks. On the surface of GO, only few layer of MoS_2 can be found and the region of few layer MoS_2 follow the shape of GO very well; (f) typical Raman spectrum taken from few layer MoS_2/GO flakes, both carbon and MoS_2 Raman signature peaks can be identified. (Figure d and e).

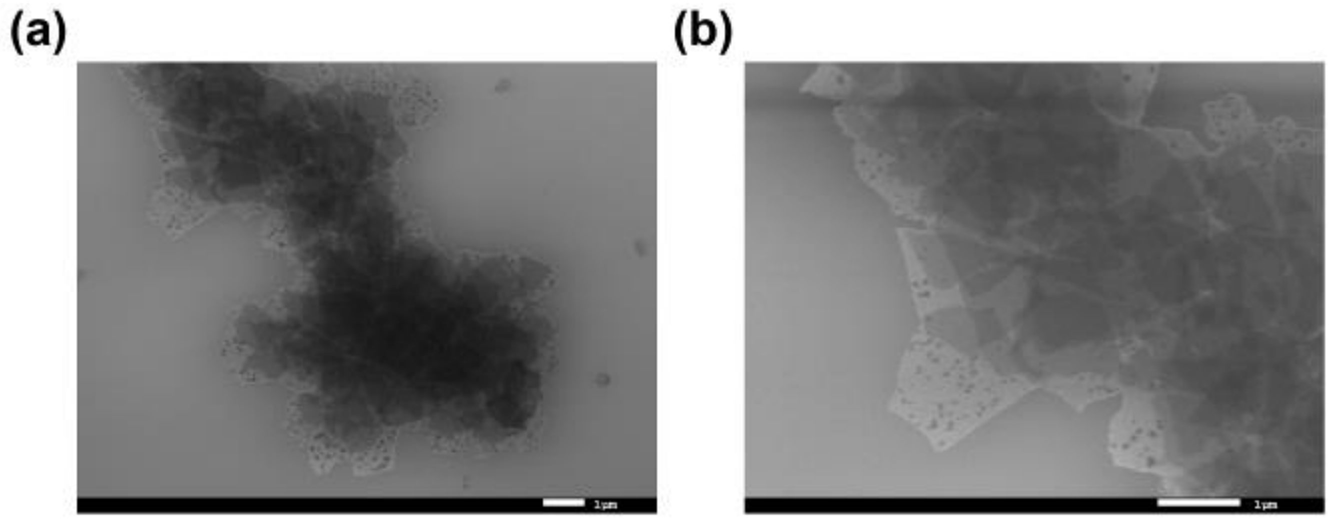

Figure S4 SEM image of staked GO layers after CVD process. The edge part shows brighter color contrast compared to the center stacked region. The isolated monolayer GO flakes (or thinner ones) tend to be etched after CVD process, which is due to the reaction of GO with MoO_3 precursor at elevated temperature.

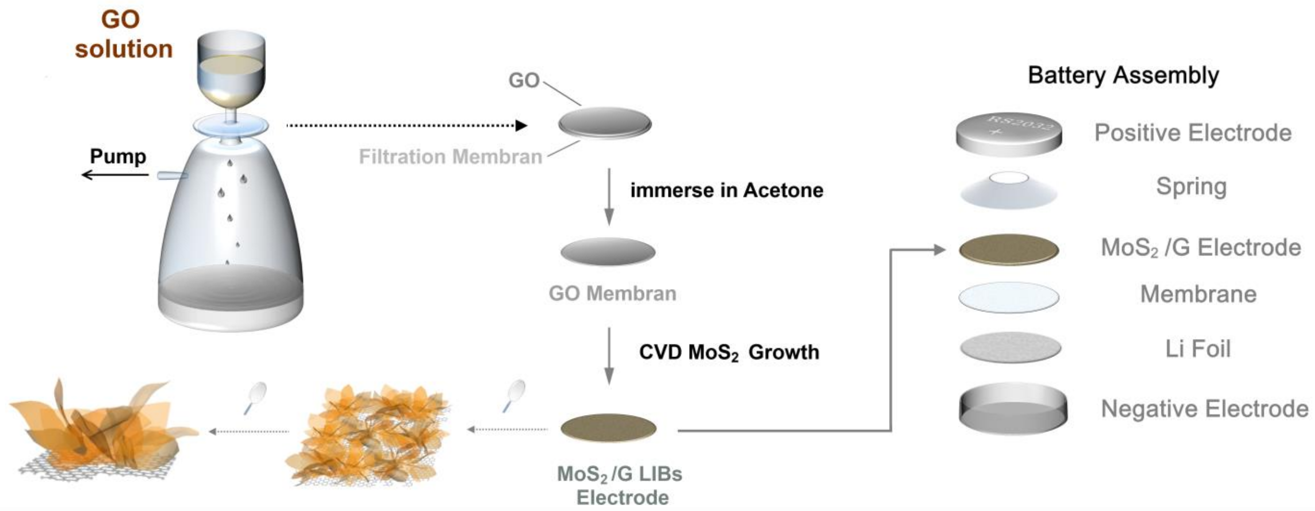

Figure S5 shows the procedure for the preparation of LIB electrodes. The active materials were directly used as working electrodes without adding any conductive binders.

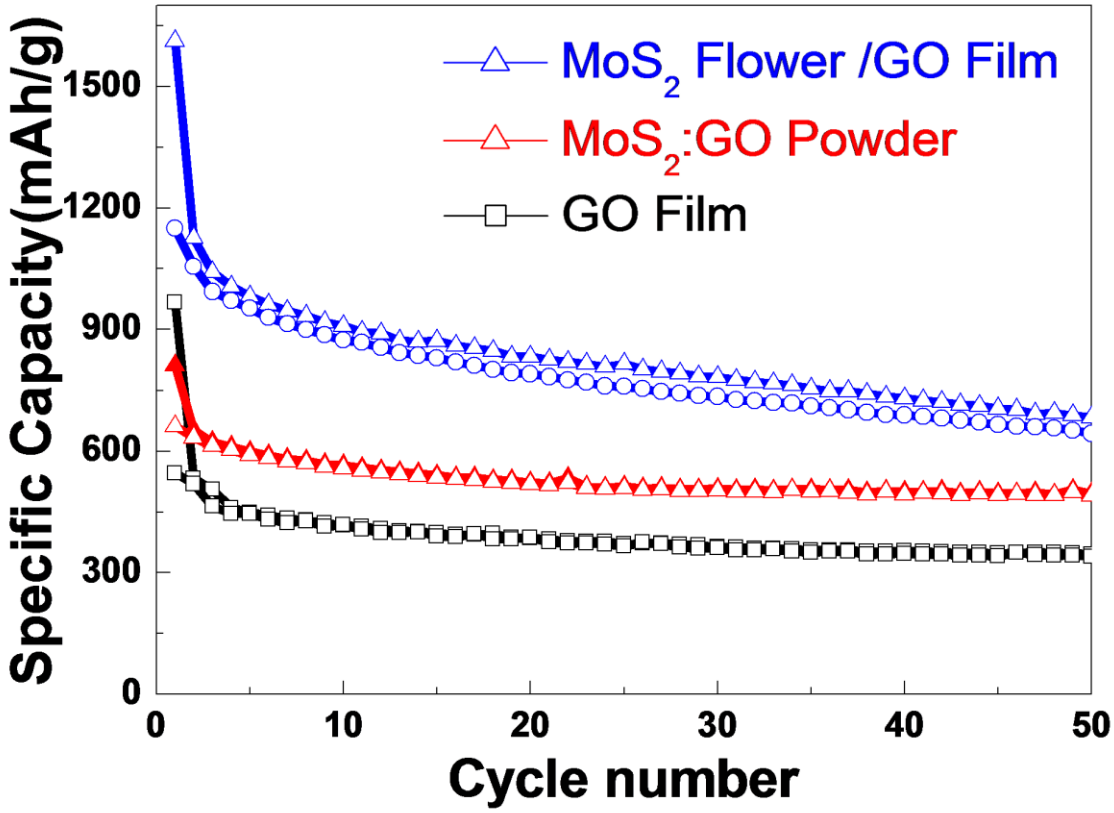

Figure S6 Comparing the discharge (Li-insertion) and charge (Li-Extraction) capacities of pure GO film, MoS_2/GO composite, and MoS_2:GO powder mixture at a current density of 100 mA g^{-1}. The theoretical reversible capacity of MoS_2 and GO are 670 and 566 mAh g^{-1}, respectively. The significantly improved charge capacity of MoS_2/GO composites could be attributed to the unique nanostructure of MoS_2 sheets on the surface of GO film. The vertical structured MoS_2 with extruding layers on GO has a much larger surface area, which provide more active sites during charging-discharging processes. The irreversible capacity loss of the 1st cycle can be mainly attributed to the electrolyte decomposition and the formation of the SEI layer. For the second cycle, a much higher Coulombic efficiency of 93.8 % was obtained and this value further increased to 95.3 % in the third cycle. After 50 cycles, a reversible capacity of 675 mAh g^{-1} can

still be retained. However, all the control samples, show a much smaller initial discharge capacity and decrease rapidly in the subsequent cycles.
